# Developing criteria and data to determine best options for expanding the core CODIS loci

**DOI:** 10.1186/2041-2223-3-1

**Published:** 2012-01-06

**Authors:** Jianye Ge, Arthur Eisenberg, Bruce Budowle

**Affiliations:** 1 Institute of Applied Genetics, Department of Forensic and Investigative Genetics, University of North Texas Health Science Center, 3500 Camp Bowie Blvd, Fort Worth, TX 76107, USA

## Abstract

**Background:**

Recently, the Combined DNA Index System (CODIS) Core Loci Working Group established by the US Federal Bureau of Investigation (FBI) reviewed and recommended changes to the CODIS core loci. The Working Group identified 20 short tandem repeat (STR) loci (composed of the original CODIS core set loci (minus TPOX), four European recommended loci, PentaE, and DYS391) plus the Amelogenin marker as the new core set. Before selecting and finalizing the core loci, some evaluations are needed to provide guidance for the best options of core selection.

**Method:**

The performance of current and newly proposed CODIS core loci sets were evaluated with simplified analyses for adventitious hit rates in reasonably large datasets under single-source profile comparisons, mixture comparisons and kinship searches, and for international data sharing. Informativeness (for example, match probability, average kinship index (AKI)) and mutation rates of each locus were some of the criteria to consider for loci selection. However, the primary factor was performance with challenged forensic samples.

**Results:**

The current battery of loci provided in already validated commercial kits meet the needs for single-source profile comparisons and international data sharing, even with relatively large databases. However, the 13 CODIS core loci are not sufficiently powerful for kinship analyses and searching potential contributors of mixtures in larger databases; 19 or more autosomal STR loci perform better. Y-chromosome STR (Y-STR) loci are very useful to trace paternal lineage, deconvolve female and male mixtures, and resolve inconsistencies with Amelogenin typing. The DYS391 locus is of little theoretical or practical use. Combining five or six Y-chromosome STR loci with existing autosomal STR loci can produce better performance than the same number of autosomal loci for kinship analysis and still yield a sufficiently low match probability for single-source profile comparisons.

**Conclusion:**

A more comprehensive study should be performed to provide the necessary information to decision makers and stakeholders about the construction of a new set of core loci for CODIS. Finally, selection of loci should be driven by the concept that the needs of casework should be supported by the processes of CODIS (or for that matter any forensic DNA database).

## Background

DNA database searching is now a fundamental tool for developing investigative leads. The purpose of a DNA database is to collect and store DNA profiles (for example, from crime scenes, offenders, or missing-persons cases) and enable comparison of the profiles. Because of recidivism, DNA databases essentially are designed to help solve future crimes. As of June 2011, searches on the Combined DNA Index System (CODIS) database have produced over 147,200 hits assisting in more than 141,300 investigations [1]. Currently, the CODIS database contains more than 10 million forensic, offender and arrestee reference profiles, and the number of profiles continues to increase. The rapid growth of the database presents the following new challenges for CODIS, as for other DNA criminal databases: 1) to address the potential of increased adventitious hits; 2) to be able to increase power for current and new applications, such as missing-persons identification and familial searching; and 3) to enable international data exchange. However, the latter may be of more limited value, for example between the US and Europe or the US and Asia. Most associations are likely to be within the country and for neighboring or open-border countries such as in Europe.

Approximately 1 year ago, the US Federal Bureau of Investigation (FBI) established a CODIS Core Loci Working Group to review and, if deemed appropriate, recommend changes to the original 13 CODIS core loci. The Working Group recently released its recommendations [2] for modification of the core loci for typing samples to be entered into CODIS. The Working Group identified 20 short tandem repeat (STR) loci (composed of the original CODIS core set minus the TPOX locus, four European recommended loci, the PentaE locus, and the DYS391 locus) plus the Amelogenin marker. These loci were placed in section A, and were recommended as the new core loci. Section B contained four additional loci that could be added in a specified order of importance, and should only be added after the loci in section A have been included in the kit(s) [2].The section A autosomal STR loci were selected from the most commonly used kits (Identifiler, PowerPlex16, and NGM SElect). Although not explained, Hares [2] placed the TPOX locus in section B, presumably because of its low power of discrimination (PD) in Caucasian populations. The loci D1S1656, D2S441, D10S1248, and D12S391 were placed in section A because they have been selected as European core loci, appear to be forensically desirable, and should enhance international data sharing. The D22S1045 locus was placed in section B, presumably because of its relatively low PD, although the reasoning was not explained by Hares [2]. The D2S1338 and D19S433 loci were placed into section A, because almost half of the profiles in CODIS contain these two loci. The PentaE locus was included in section A, owing to its 'high discrimination value, low mutation rate, and usefulness in mixture deconvolution' [2]. By contrast, the PentaD locus was placed in section B. No explanation was given for this, yet the PentaD locus has a match probability that is better than 12 of the loci in section A (Table 1). Although highly polymorphic, the SE33 locus was included in section B, mostly likely due to its high mutation rate. Including one Y-chromosome STR (DYS391) is one of the more interesting recommendations of these newly proposed core loci. Hares [2] stated that the DYS391 locus was selected to 'confirm Amelogenin null values sometimes present in DNA typing.' The reported motivations for selecting a revised core set were: 1) to reduce the likelihood of adventitious matches because the CODIS database has, and will continue to substantially increase in size; (2) to increase international compatibility for better data sharing; and (3) to increase discrimination power for missing-persons cases.

**Table 1:** General information on the STR loci selected by Hares [2], including chromosomal location, loci in kits or panels, mutation rates, and match probabilities, based on a Caucasian population^1-3^

Locus	**Panels/Kits**						Location	Size, Mb	Mutation rate		MP^7^
						
	13 Core loci	New FBI core loci^4^	European loci^5^	Identifiler	PowerPlex 16	China^6^			Paternal	Maternal	
D1S1656		A	S				1q42	228.972	1.54 × 10^-3^	3.70 × 10^-4^	0.019
D2S441		A	S				2p14	68.214	1.54 × 10^-3^	3.70 × 10^-4^	0.095
D2S1338		A	D	√			2q35	218.705	1.36 × 10^-3^	2.49 × 10^-4^	0.028
D3S1358	√	A	S	√	√	√	3p21.31	45.543	1.68 × 10^-3^	2.55 × 10^-4^	0.076
FGA	√	A	S	√	√	√	4q28	155.866	3.71 × 10^-3^	4.93 × 10^-4^	0.038
D5S818	√	A		√	√	√	5q23.2	123.139	1.66 × 10^-3^	2.69 × 10^-4^	0.158
CSF1PO	√	A		√	√	√	5q33.1	149.436	1.98 × 10^-3^	3.19 × 10^-4^	0.118
D7S820	√	A		√	√	√	7q21.11	83.433	1.37 × 10^-3^	7.23 × 10^-5^	0.065
D8S1179	√	A	S	√	√	√	8q24.13	125.976	2.06 × 10^-3^	3.33 × 10^-4^	0.061
D10S1248		A	S				10q26.3	130.567	1.54 × 10^-3^	3.70 × 10^-4^	0.092
TH01	√	A	S	√	√		11p15.5	2.149	5.20 × 10^-5^	6.03 × 10^-5^	0.081
D12S391		A	S				12p13.2	12.215	1.54 × 10^-3^	3.70 × 10^-4^	0.02
VWA	√	A	S	√	√	√	12p13.31	5.963	3.25 × 10^-3^	4.68 × 10^-4^	0.063
D13S317	√	A		√	√	√	13q31.1	81.620	1.74 × 10^-3^	4.03 × 10^-4^	0.085
PentaE		A			√		15q26.2	95.175	2.60 × 10^-4^	2.53 × 10^-4^	0.02
D16S539	√	A	D	√	√	√	16q24.1	84.944	1.03 × 10^-3^	5.25 × 10^-4^	0.1
D18S51	√	A	S	√	√	√	18q21.33	59.100	2.23 × 10^-3^	7.93 × 10^-4^	0.029
D19S433		A	D	√			19q12	35.109	9.75 × 10^-4^	5.48 × 10^-4^	0.088
D21S11	√	A	S	√	√	√	21q21.1	19.476	1.75 × 10^-3^	1.18 × 10^-3^	0.046

DYS391		A					Yq11.21	14.103	1.70 × 10^-3^	-	0.455

TPOX	√	B		√	√		2p25.3	1.472	1.65 × 10^-4^	1.05 × 10^-4^	0.195
SE33		B	D				6q14	89.043	6.40 × 10^-3^	3.00 × 10^-3^	0.005
PentaD		B			√		21q22.3	43.880	2.59 × 10^-4^	2.53 × 10^-4^	0.049
D22S1045		B	S				22q12.3	35.779	1.54 × 10^-3^	3.70 × 10^-4^	0.134

Loci, n	13	20 + 4	12 + 4	15	15	11					

^1^Amelogenin is not included.

^2^'√' means that the locus is in the particular panel.

^3^The table is sorted by chromosome and location, and sections A and B loci are separated.

^4^'New FBI core' refers to the panel described by Hares [2]. 'A' and 'B' denote the loci placed into sections A and B, respectively.

^5^'S' denotes the loci in European Standard Set (ESS); 'D' denotes additional loci to expand the European Standard Set. The European loci panel differs by one locus (SE33) from the NGM loci [7].

^6^Eleven STR loci common to five major commercial kits used in China: Identifiler, Sinofiler, PowerPlex16, DNAtyper15, and AGCU (17+1) [24].

^7^MP, match probability.

The consideration of expanding and/or replacing the core loci is lauded. The CODIS system should be reviewed on a routine basis to improve capabilities and efficiencies with database searches. However, Hares and his Working Group [2] provided limited or no data or justifications for their selections. Indeed, some of the recommendations seem to be in conflict with the selection criteria originally defined by the Working Group. The purpose of expanding and deselecting CODIS core loci was to respond to current and projected challenges and improve performance to meet the needs of forensic applications. However, based on the selections, the resultant choice of loci may not provide the optimum performance of such a DNA database. Countries interested in establishing a DNA database and selecting their core loci might wish to proceed with caution if using the model described by Hares [2]. Given the selection process described by Hares [2], it is worth asking whether the needs of CODIS should drive casework requirements, or the needs of casework should drive CODIS requirements. We believe the latter position is the correct one to take; however, the selection of new FBI CODIS core loci seems to be a greater reflection of the former position. The quality of casework evidence will always be the limiting factor and should be a primary driver for selecting loci. In addition, the power of the set of loci should be evaluated with regard to the number of potential hits for a given the application (for example, direct single source, mixtures, and indirect familial searching) and database size. The primary application of the CODIS database has been to search for the 'single source match' in the database, and most investigation leads fall into this category. The national level of CODIS (National DNA Index System; NDIS), requires that a forensic profile should contain a minimum of 10 loci. This allowance for fewer than 13 loci for forensic samples is a clear recognition that forensic DNA can be compromised, and full profiles are not always obtainable. Fewer than 10 loci are not permitted for upload to avoid generating too many adventitious hits. NDIS does accept additional loci beyond the 13 core loci, but currently does not use these loci in the initial search parameters. Currently, NDIS only accepts mixture profiles that meet the '4 by 4 rule' (that is, a forensic profile can have up to 4 alleles at a maximum of four core loci and no more than 2 alleles at any of the remaining 9 core loci, or 6 loci if only the minimum of 10 loci is submitted) [3]. Hares [2] did not describe whether the '4 by 4 rule' (better described as a 9 by 2 rule) would still apply if the new core loci are adopted. Currently, it is assumed that the rule will continue, probably because the selection of new loci does not seem to account for the effect on quality and quantity of DNA derived from forensic samples. The criteria for additional loci should be considered as they apply to single-source data, mixture results (if this is a required search condition), and projected kinship applications given a database of size N. Although it is obvious that adding more loci in a virtual sense will increase power, changes to the CODIS core loci first should be based on the power and efficiency of the current loci, and equally as important (if not more so), whether they meet the needs of forensic applications. If they do not, then the alternative loci that would be most applicable to those needs should be selected. For example, the TPOX locus was relegated to the second-tier level, and although we agree with this based on the PD, the TPOX locus may in fact perform much better in casework than more informative loci, such as the FGA locus. The FGA locus is a large amplicon locus and is more likely to drop out with degraded or inhibited samples compared with the TPOX locus (at least for some kit configurations). Even when the amplicon size of the TPOX and FGA loci overlap, the wider spread of alleles for the FGA locus yields greater heterozygote peak height imbalance and allele dropout than the TPOX locus (and other STR loci), particularly for challenged samples. It does not appear that locus performance in casework analyses was taken into account during selection of the chosen loci [2]; if a locus in a compromised sample cannot be typed, it cannot be uploaded to a database. The selection of core loci is therefore more complex than just determining what loci are available, and most importantly, the needs of casework should be considered in the selection process.

The criteria that the Working Group [2] used to base its selection of core loci are: 1) No known association with medical conditions or defects (refers to whether there is a reported association of the locus with a medical condition or disease status); 2) low mutation rate (a locus with a mutation rate preferably of less than 0.30%); 3) high level of independence (refers to linkage equilibrium (LE) of the loci on the list to enable multiplication of genotype frequencies); 4) high level of discrimination (a locus with a probability of identity preferably of less than 0.10%) (note: this value is obviously a typographical error, and is more likely to be 0.1); 5) use by the international forensic DNA community (refers to the use - widespread or limited - of the loci by forensic DNA laboratories outside the USA); 6) number of loci versus discrimination factor (refers to balancing the total number of loci recommended with the level of discrimination they offer); 7) compliance with quality assurance standards (refers to the loci satisfying the requirements of the FBI Director's Quality Assurance Standards such as validation, being human-specific, etc.).

These are reasonable criteria, except for the omission of the potential effect on test performance with DNA degradation and inhibition. However, no systematic and scientific assessments of the selected loci, or how they comport with the selection criteria, were described. Additionally, the selection process did not provide any data on a number of issues, such as the power of the current core loci and the projected database sizes, the limitations invoked by the quality of casework materials, the perceived need for resolving Amelogenin Y-amplicon drop-out when searching for candidates, the justification of suggesting the low PD Y-STR locus DYS391 (particularly given the downgrading of the TPOX locus because of its low PD), alternative applications (for example, familial searching and missing-persons identification), and the reduction in sensitivity of detection that can occur if multiplexes become larger.

In this paper, we provide examples and simplified analyses as potential considerations while the community moves forward in modifying the CODIS core loci and for countries that are currently instituting DNA databases. We did not attempt to address all criteria in depth. Instead, we analyze and discuss the issues with examples to make the point that selection is a more complex process than Hares [2] seems to have taken into account, and the process should be given more in-depth consideration with wider community input. Indeed, the European Network of Forensic Science Institutes (ENFSI) used input from its multi-country members to produce a consensus-built standard clearly driven by the demands of typing challenged samples (supported by selecting a number of mini-STRs [4-6]). The examples provided in this paper are simplified analyses on the performance of various combinations of STR loci for their adventitious hit rates in reasonably large datasets for the primary forensic applications of single-source profile comparisons, mixture comparisons, and kinship searches, and for international data sharing. These examples could provide a basis for the issues to consider and the work that might be performed to support core STR loci selection criteria.

## Methods and Results

### Data sources

We obtained the allele frequencies of the Caucasian population for the loci D10S1248, D12S391, D16S539, D18S51, D19S433, D1S1656, D21S11, D22S1045, D2S1338, D2S441, D3S1358, D8S1179, FGA, TH01, and vWA from Budowle *et al. *[7], for D13S317, D7S820, D5S818, CSF1PO from Budowle *et al. *[8], for DYS391 from Budowle *et al. *[9], PentaD and PentaE from Budowle *et al. *[10], and for SE33 from Butler *et al. *[11]. The mutation rates in Caucasian populations for the loci CSF1PO, FGA, TH01, TPOX, VWA, D3S1358, D5S818, D7S820, D8S1179, D13S317, D16S539, D18S51, D21S11, D2S1338, D19S453, PentaD, and PentaE were taken from the American Association of Blood Banks (AABB) annual report for 2008 [12], and those for the SE33 locus from STRBase [13].The mutation rates of the loci D10S1248, D12S391, D1S1656, D2S441, and D22S1045 were not available, thus their rates were assigned as the average of the tetranucleotide markers. The mutation rates of 16 Y-STR loci of Caucasian and world population data were from Ge *et al. *[14] and YHRD [15], respectively. Chromosomal locations of the STR loci were from NCBI [16] and STRBase [13] (Table 1).

### Evaluation of the autosomal STR loci

#### Independence between loci

Current autosomal STR-based forensic applications assume independence between the core CODIS STR loci, so that the match probability, kinship index (KI), or likelihood ratio (LR) of each locus can be multiplied together. The community seems to favor independent loci, except where this is not possible, such as the lineage markers on the Y chromosome and the mitochondrial DNA (mtDNA) genome. The desire for independent autosomal STR loci is presumably due to the ease of calculation compared with a more complicated estimation of haplotype frequencies. We do not comment on the position of selecting independent loci; we merely acknowledge it and note that it was a criterion of the Working Group. It is likely that using systems that are relatively independent is easier for the community, and there are sufficient STR loci to select ones that meet the criterion of biologic independence.

Independence between loci usually requires that the loci are not genetically linked and that they are in LE. However, Hares [2] set a criterion of LE but seemed to neglect genetic linkage between the loci. This misunderstanding was also espoused by O'Connor *et al. *[17], although those authors later provided a correction [18]. LE between the alleles at two loci may sometimes be met at the population level, and the loci can be assumed to be independent for direct single-source and mixture comparison calculations without corrections [7]. However, genetic linkage describes the situation where loci that are physically close to each other tend to be inherited together in families. Genetic linkage, measured by recombination fraction, should be considered before assuming independence for kinship analyses. Ideally, recombination should be close to 50% for the assumption that two loci are unlinked, and can be used independently in kinship analysis. The loci VWA and D12S391 do not significantly deviate from LE at the population level; however, they reside on the same chromosome about 6 Mb apart, and the recombination fraction is approximately 11%. The data indicate that the KIs of the VWA and D12S391 loci cannot be directly multiplied together [7]. Indeed, these two loci do not meet the Working Group's third criterion regarding independence, that is, 'High level of independence (refers to linkage equilibrium of the loci on the list to enable multiplying genotype frequencies)' or the motivation 'to increase discrimination power to aid missing persons cases [2]'. As can be seen by the chromosome locations of the 24 STR loci (Table 1), in addition to the VWA and D12S391 loci, the distance between the D5S818 and CSF1PO loci and the D21S11 and PentaD loci are about 26 Mb and 24 Mb, respectively. These additional two pairs may also be genetically linked. Phillips *et al. *[19] described, with reasonable assumptions, recombination between the loci D5S818 and CSF1PO and between D21S11 and PentaD of 25.22% and 35.68%, respectively, based on HapMap data [20]. Family-based linkage studies should be carried out to confirm the recombination fractions before selecting core loci that meet the criterion of independence. The effect of close linkage on forensic applications should be investigated further. We do recognize that it may not be possible to satisfy all desired criteria; however, given the large battery of available loci, there is no need to compromise the 'independence criterion' for autosomal STRs if building a better (that is, more informative) system for the future growth of databases is desired.

#### Single-source profile comparison

The primary application in CODIS searches is single-source profile comparisons. Discrimination power or match probability (MP) of the current and proposed STR loci should be evaluated. Most autosomal loci have MP values of less than 0.1 (Table 1). The SE33 and D1S1656 loci have the lowest MP (that is, are the most informative) of all loci in sections A and B. The TPOX locus has the highest MP (that is, is the least informative) of all the autosomal loci listed by Hares [2]. The D22S1045 locus has the highest MP among the European loci. Based on PD or MP, these two loci seem to be better suited to section B as the Working Group recommended [2].

We calculated the expected MP (EMP) of the current multiplex kits or panels of loci using previously described methods [21,22] for unrelated, full-sibling, and parent/child relationships (Table 2). Caucasian population data were used as an example (such analyses also need to be carried out on other relevant populations by the Working Group; the examples given here are from one population for illustrative purposes). With 13 CODIS core loci, the chance of generating adventitious matches between unrelated people for single-profile searches in a database (that is, 1 to N) is extremely low. For example, for a database of 100 million profiles (N = 10^8^, which is a database an order of magnitude larger than the current size of CODIS), the EMP is 1 in 10 million (based on a random match probability of 10^-15^). Thus, the chance of multiple hits in a large database is exceedingly small. The population substructure effect can increase the EMP by roughly 2 to 10 times depending on the number of loci, but would not substantially change the ability of the current 13 core loci to meet the need for single-source profile comparisons. Thus, single-source profile searches are well met by the current core 13 STRs, and facilitated by the currently available kit configurations. The EMP will increase significantly among relatives (even those in a database), but most people do not have such large families that adventitious full-profile hits from relatives would become unmanageable. Recall that database searches generate investigative leads and two, or even three, matching single-source profiles are certainly tolerable.

**Table 2:** The expected match probability (EMP) of the kits/panels.^1^

Panel (number of STR loci)	Unrelated		Parent/child		Full sibling	
	
	Fst = 0^2^	Fst = 0.01	Fst = 0	Fst = 0.01	Fst = 0	Fst = 0.01
New FBI core (24)^3^	6.28 × 10^-30^	5.12 × 10^-29^	3.63 × 10^-18^	1.15 × 10^-17^	3.49 × 10^-11^	4.86 × 10^-11^
New FBI core section A (20)^3^	9.54 × 10^-25^	4.77 × 10^-24^	3.83 × 10^-15^	9.37 × 10^-15^	1.74 × 10^-9^	2.29 × 10^-9^
13-loci CODIS core (13)	2.34 × 10^-15^	5.83 × 10^-15^	1.74 × 10^-9^	2.86 × 10^-9^	3.39 × 10^-6^	4.05 × 10^-6^
Identifiler (15)	5.93 × 10^-18^	1.73 × 10^-17^	5.04 × 10^-11^	9.17 × 10^-11^	4.21 × 10^-7^	5.17 × 10^-7^
PowerPlex16 (15)	2.43 × 10^-18^	7.48 × 10^-18^	3.06 × 10^-11^	5.74 × 10^-11^	3.61 × 10^-7^	4.45 × 10^-7^
NGM^4 ^(15)	1.12 × 10^-19^	4.15 × 10^-19^	5.68 × 10^-12^	1.17 × 10^-11^	2.03 × 10^-7^	2.52 × 10^-7^

^1^Caucasian population data were used.

^2^Fst is the autosomal short tandem repeat (STR) co-ancestry coefficient for population substructure correction. ^3^The DYS391 locus is included only in 'New FBI core' and 'New FBI core section A', and no population substructure correction was applied to this locus.

^4^NGM has the same STR loci as the 'European loci' excluding the SE33 locus.

One essential criterion not addressed by the Working Group or described by Hares [2] is what may be considered 'manageable'. There should be some discussion on the number of associations per search that can be tolerated, as this will assist in determining the power needed. This concept of manageability is not a simple one to address, but obviously has an effect on performance goals.

Even with the larger databases that are expected in the near future, the current battery of loci provided in already validated commercial kits (for example, Identifier, PowerPlex16, and NGM) meet the needs for single-source profile comparisons, including those with a significant proportion of relatives and subpopulations. Adding more loci in a virtual sense will increase the PD, but on a practical level, little efficiency is gained for single-source comparisons even for a database containing more than 100 million reference profiles.

#### Kinship analysis

One of the reasons proffered by Hares [2] to expand the CODIS core loci is to aid missing-persons identification, which also in turn would facilitate partial match (the result of moderate stringency searches) and familial searching. These functionalities of missing-persons identification and familial searching employ kinship analysis. The best loci for kinship analysis are loci that have low mutation rates, are independent, and have high average KI (AKI), a measure similar to MP in single-source profile comparison. The lack of independence between some of the loci in section A has been addressed above. The SE33 locus is highly polymorphic and is particularly discriminating for direct comparisons. However because of its high mutation rate, SE33 is a poor locus for kinship analysis (and this is probably the reason that the SE33 locus was relegated to section B and may indicate that Hares [2] favored the criterion of low mutation rate over superior MP). To evaluate the informativeness of the loci, the AKI of each locus in section A was calculated by simulation (as described previously [22,23]; note that the software used (MPKin) is being developed into a user-friendly format, and once developed, will become commercially available) for full-sibling and parent/child relationships with Caucasian population data (Table 3). The AKI rank by locus is similar to the MP rank in Table 2. In section A, D1S1656, D12S391, and PentaE are the most informative loci, and D5S818 is the least informative locus. The AKI values of the loci D22S1045 and TPOX are lower than those of most of the other loci.

**Table 3:** Average kinship index (AKI) of the short tandem repeat (STR) loci for full-sibling (FS) and parent/child (PC) relationships with Caucasian population data.^1,2^

Locus	AKI	
	
	PC	FS
PentaE	3.47	2.74
D12S391	3.37	2.75
D1S1656	3.37	2.67
D2S1338	2.91	2.41
D18S51	2.84	2.38
FGA	2.63	2.23
D21S11	2.54	2.18
D8S1179	2.31	2.05
D7S820	2.09	1.84
VWA	2.08	1.85
D19S433	2.07	1.93
D13S317	2.00	1.81
D2S441	1.99	1.76
D3S1358	1.92	1.71
D10S1248	1.88	1.74
D16S539	1.87	1.73
TH01	1.85	1.68
CSF1PO	1.79	1.64
D5S818	1.65	1.55

DYS391	2.20	2.20

SE33	6.24	4.42
PentaD	2.32	2.04
D22S1045	1.74	1.63
TPOX	1.58	1.47

^1^The AKI values were estimated by 100,000 simulations for each locus.

^2^This table is sorted by the AKI of parent-child relationship for section A loci (excluding the DYS391 locus) and section B separately.

**Figure 1:**
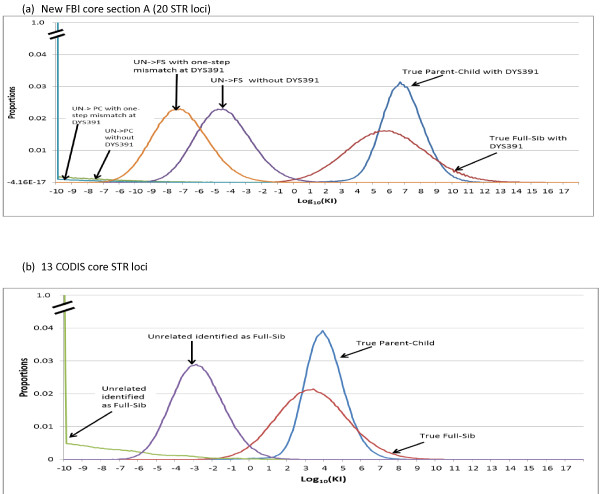
**The log_10 _of the kinship index (KI) distributions for parent/child (PC) or full-sibling (FS)**. Log_10_(KI) distributions for parent/child (PC) or full-sibling (FS) identified as unrelated profiles and unrelated identified as potential related profiles. **(A) **The new FBI core loci in section A (20 STR loci); **(B) **the 13 current CODIS core loci. In total, 1 million simulations were performed for each distribution. The KI of the DYS391 locus for true relatives is 2.2. The distributions of true parent/child distributions with or without the DYS391 locus are close, as were the true full-sibling distributions.

In addition, we performed simulations to generate KI distributions of the section A and 13 CODIS core loci for unrelated profiles identified as deriving from full-sibling or parent/child (Figure 1). The KI of the DYS391 locus for true relatives is the inverse of the MP of the DYS391 locus (that is, 1 ÷ 0.455 = 2.2). The KI of the DYS391 locus for unrelated people with a one-step mismatch is close to the mutation rate of the locus (that is, 0.0021). With 13 CODIS core loci, a large proportion of relatives will not be resolved from unrelated candidates, especially for the full-sibling relationship. With a KI of 1,000 or greater, there are about 5% parent/child and 40% full-sibling pairs that would be excluded as unrelated in a database search. Adding two more loci (for example, those in Identifier or PowerPlex16) can provide better performance [22,23]. If all 20 loci in section A are used (these are assumed independent for illustrative purposes even though some do not meet this criterion), less than 1% of true parent/child and about 15% of true full-sibling associations would be excluded. The 13 CODIS core loci are not sufficiently powerful for kinship analyses, and 20 or more autosomal STR loci do perform better (however see below on the effect of Y-STRs for kinship-analysis performance).

#### Mixture profiles

Mixture profiles are very common in casework and are likely to increase as more high-volume crime evidence is subjected to DNA typing. Currently, the CODIS upload criteria preferentially selects for single-source profiles, and thus mixtures are not of great concern. However, to increase the number of developed investigative leads, the effect of mixtures should be considered when selecting core loci and in the context of how they are accommodated for uploading and searching within CODIS. Multiple potential contributors to a mixture profile may be found in a database search. The goal should be that the number of potential contributors should be small and manageable for investigative purposes (we note as stated above that the term 'manageable' has not been defined by the Working Group and this is something that perhaps should be addressed prior to evaluating the power of the loci).

**Figure 2:**
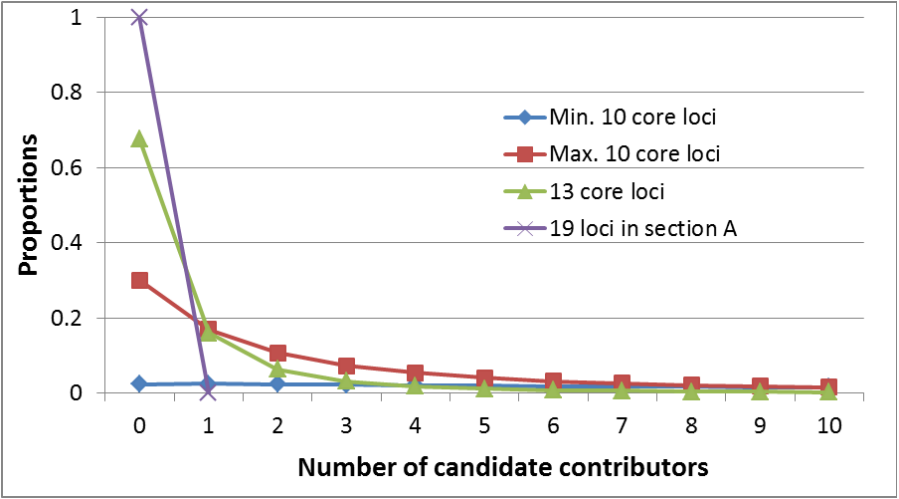
**The distributions of the number of included profiles in a two-person mixture based on autosomal STRs for four panels**. The four panels were the 13 CODIS core loci, the 19 autosomal loci in section A, the 10 most informative of the 13 CODIS core loci (D18S51, FGA, D21S11, D8S1179, VWA, D7S820, D3S1358, TH01, D13S317, and D16S539), and the 10 least informative of the 13 CODIS core loci (D8S1179, VWA, D7S820, D3S1358, TH01, D13S317, D16S539, CSF1PO, D5S818, and TPOX). The distributions were obtained by simulation, in which 1 million profiles were first generated as a database, and then 1 million two-person mixtures were randomly generated without replacement. Each mixture was searched against the database to determine the number of candidate part-contributors beyond those that comprised the mixture. The Y-axis represents the proportion of mixtures with specific number of candidate contributors in a database search. For example, with 13 CODIS loci, no candidate contributors were identified for 67.7% of mixtures. Only 1.3% or 0.4% of two-person mixtures generated more than 10 or 20 candidate contributors, respectively. With the 19 loci in section A, almost 100% of two-person mixtures had no candidate contributors in a database of 1 million profiles.

We calculated (Figure 2) the distributions of the number of candidate contributors in two-person mixtures based on autosomal STRs and searching a database of 1 million profiles for 4 panels (the 13 CODIS core loci, the 19 autosomal loci in section A, and the 10 most informative and 10 least informative loci of the 13 CODIS core loci). The adventitious candidate contributors are the profiles found in a database beyond the 2 true contributors comprising the mixture (that is, the hits in the database search without replacement). With 13 CODIS loci, no candidate contributors were found for 67.7% of mixtures. Only 1.3% or 0.4% of the 2-person mixtures generated more than 10 or 20 candidate contributors, respectively (Figure 2). If all autosomal loci in section A were included, almost no 2-person mixtures generated candidate contributors in a database search of 1 million profiles.

It seems that, in a database with 1 million profiles, most 2-person mixtures will yield a small number of candidate contributors with 13 CODIS loci. With additional loci included in the core set, fewer candidate contributors are expected from a search with a mixture profile. For a database with 10 million or more profiles, the distributions are expected to move towards an increased number of candidate contributors. More precise distributions for larger databases can be obtained with more powerful computational resources.

#### International data sharing

International data sharing across countries is another reason espoused to expand the CODIS core loci [2]. This criterion is more important for neighboring and open-border countries, such as those in Europe. The number of anticipated hits between, for example, Europe and the USA, is expected to be very few compared with all within-country searches. Thus, the requirement for international compatibility may not be as important as other selection criteria. The USA might be better served by ensuring compatibility with Canada and Mexico. More data are needed on the expected number of searches between the US and other areas such as Europe, Asia, and Latin America to determine the effect of compatibility. Regardless, most data sharing focuses mainly on single-source profile comparisons, and the 13 CODIS core loci share 7 loci in common with the European Standard Set ('S' in Table 1) and 8 loci in common with the new European Standard Set (including both 'D' and 'S' in Table 1). The EMPs of the shared 7 and 8 loci are 1.2 × 10^-9 ^and 1.2 × 10^-10^, respectively, which on average seems to be practical for data exchange with current and larger sized databases, as the number of adventitious associations is expected to be low for single-source profile comparisons. However, a large proportion of the database profiles (in the USA and presumably in other countries) also contain the loci D2S1338 and D19S433, thus the EMP reduces to 10^-13^. Adding D1S1656, D2S441, D10S1248 and D12S391 (also which are generally well suited to typing relatively degraded samples) to the core loci, as Hares [2] suggested, can reduce the EMP to 10^-16^, but on a practical level, international data sharing with European databases may not need these additional loci.

China has the single largest forensic DNA database, which currently contains almost 12 million profiles. There was no discussion by Hares [2] on compatibility with China. There are five major commercial kits used in China, among which 11 loci (see Table 1) are shared by these five predominant commercial kits [24]. These 11 loci are all within the current CODIS core loci. The EMP of these 11 loci can reach 1.6 × 10^-13 ^and 1.5 × 10^-13 ^for Chinese Han and Caucasian populations, respectively, and is sufficiently low for data sharing between China and the USA. These 11 loci include 6 loci in common with the European Standard Set and 7 loci with the new European Standard Set, with EMPs of 1.5 × 10^-8 ^and 1.5 × 10^-9^, respectively. China will continue to move forward and formalize its core set of loci, and perhaps compatibility with those loci should be considered. Regardless, there may be sufficient compatibility for most single-source searches between the US and China.

There are two points that we do not address here, which should be considered before selecting loci: 1) many adventitious matches can be excluded by non-genetic information and thus, how that information would be used with the genetic data should be explored for practicality; and 2) the percentage of cases that would be facilitated by international sharing should be assessed. Most crimes will occur within a country or bordering countries. Although we personally do not have the data to resolve the value of international sharing, the utility of this should be considered.

### Evaluation of Y-chromosome short tandem repeats

Y-STR loci are very useful in forensic investigations because they can be used to trace paternal lineage, deconvolve female and male mixtures, and resolve inconsistencies with Amelogenin typing (although Amelogenin is not used routinely in direct comparison single source and mixture CODIS searches). Most profiles in CODIS are from men, thus Y-STR data are particularly useful for discriminating between the donor profiles in CODIS. Hares [2] recommended use of the DYS391 locus to resolve Amelogenin discrepancies; however, CODIS does not use Amelogenin for searching. In addition, the DYS391 locus is one of the least informative Y-STR loci compared with other Y-STRs (Table 4) in Caucasian and other major populations [25]. The MP of the DYS391 locus is around 0.45, thus, the DYS391 locus is clearly less discriminating than even the TPOX locus. Perhaps this locus was chosen because it could be accommodated in a small amplicon or because there were few null alleles in the population. However, taking up valuable multiplex space with this locus makes little practical sense, especially as Amelogenin is not routinely used for searching. There are many Y-chromosome STR loci that are more informative and could be placed in a multiplex. The DYS385 locus is apparently the most informative forensically relevant Y-STR locus because it is the result of a tandem duplication; thus it provides an upper bound on the PD of a single Y-STR locus. The KI for the DYS385 locus is 2.6 times higher (for the Caucasian population) than that of the DYS391 locus, which is comparable with one very informative autosomal STR. Moreover, the DYS385 locus has a relatively low mutation rate, even slightly lower than the DYS391 locus (Table 4). We are not suggesting particularly that the DYS385 locus be selected over other Y-STR loci; that choice should be made based on the performance criteria as stressed in the study herein. We merely point out that if selection of only a single Y-STR locus was the best choice, there are Y-STRs available that are more informative but can still be placed within a small amplicon. However, the allele spread of the allelic ladder for the DYS385 locus is 7 to 25 repeats, which could be accommodated in a smaller amplicon [16,26], and this capability would have to be balanced with the degree of differential amplification of 'heterozygous' alleles.

**Table 4:** Match probability and mutation rates per Y-STR locus.

Locus	MP	Mutation rates × 10^-3^	
		
		Caucasian	YHRD
DYS385	0.17	1.57	2.134
DYS458	0.23	1.05	6.444
DYS456	0.27	8.36	4.243
DYS389II	0.3	1.04	3.644
DYS390	0.31	1.05	2.102
DYS439	0.35	0	5.214
DYS635	0.37	3.13	3.467
DYS448	0.37	2.09	1.571
DYS392	0.4	0	4.123
YGATA H4	0.41	2.09	2.434
DYS437	0.41	2.09	1.226
DYS438	0.42	0	3.059
DYS391	0.45	2.09	2.599
DYS19	0.46	0	2.299
DYS389I	0.48	1.05	2.523
DYS393	0.68	2.09	1.045

The table is sorted by increasing match probability (MP), shown in the second column.

The column 'YHRD' lists the mutation rates from http://www.yhrd.org/[15]; all other match probability and mutation rates were from Budowle *et al *[9] and Ge *et al *[14].

For Y-STR haplotypes with all 16 Y-STR loci (in the YFiler kit) the MP can reach 0.0011 (Table 5), which is comparable with the cumulative MP of the 3 least informative autosomal STR loci in section A, although the MP of a Y-STR haplotype depends on the population and the database size. It is not efficient to include all 16 Y-STR loci rather than a few autosomal loci in the new CODIS core loci, either from a PD or multiplex practicality point of view. The MP of two autosomal STR loci is comparable with six of the most informative Y-STR loci haplotypes (about 0.003). By contrast, for kinship analysis, the KI of relatives for the six most informative Y-STR loci haplotypes (KI = 372) is comparable with 5 or 6 of the most informative autosomal STR loci in section A (that is, AKI = 392 with the top 5 loci for parent/child or AKI = 410 with the top 6 loci for full-sibling relationships in section A; Table 3), or similar to 9 or 10 of the least informative autosomal STR loci (that is, AKI = 301 with the bottom 9 loci for parent/child or AKI = 456 with the bottom 10 loci for full-sibling relationships in section A; Table 3). In addition, the 6 most informative Y-STR loci can exclude 99.7% of unrelated profiles, whereas the 6 most informative autosomal STR loci can only exclude about 90% or 99% unrelated candidates as full siblings or parent and offspring, respectively, even with maximum accuracy thresholds.

**Table 5:** Y- chromosome short tandem repeat (Y-STR) combinations with minimum match probability (MP) for a specified number of Y-STR markers.^1^

Number of loci	Y-STR combinations with minimum MP^2^	MP	KI = 1/MP^3^
1	15	0.1748	5.72
2^4^	5, 15	0.0477	20.95
3	3, 5, 15	0.0178	56.25
4	1, 3, 5, 15	0.0083	121.1
5	1, 2, 3, 5, 15	0.0045	223.65
6	1, 2, 3, 5, 13, 15	0.0027	372.45

7	1, 2, 3, 5, 9, 13, 15	0.0020	501.29
8	1, 2, 3, 5, 9, 13, 14, 15	0.0016	620.91
9	1, 2, 3, 5, 9, 10, 13, 14, 15	0.0014	711.62
10	1, 2, 3, 5, 8, 9, 10, 13, 14, 15	0.0013	770.27
11	1, 2, 3, 4, 5, 8, 9, 10, 13, 14, 15	0.0012	819.92
12	0, 1, 2, 3, 4, 5, 8, 9, 10, 13, 14, 15	0.0012	847.22
13	1, 2, 3, 4, 5, 6, 8, 9, 10, 11, 13, 14, 15	0.0012	866.46
14	0, 1, 2, 3, 4, 5, 6, 8, 9, 10, 11, 13, 14, 15	0.0011	876.41
15	0, 1, 2, 3, 4, 5, 6, 8, 9, 10, 11, 12, 13, 14, 15	0.0011	886.59
16	0, 1, 2, 3, 4, 5, 6, 7, 8, 9, 10, 11, 12, 13, 14, 15	0.0011	891.77

^1^This table was generated from the data of Budowle *et al *[9].

^2^0 = DYS389I, 1 = DYS389II, 2 = DYS390, 3 = DYS456, 4 = DYS19, 5 = DYS458, 6 = DYS437, 7 = DYS438, 8 = DYS448, 9 = Y GATA H4, 10 = DYS391, 11 = DYS392, 12 = DYS393, 13 = DYS439, 14 = DYS635, and 15 = DYS385.

^3^Kinship index (KI) for true paternal lineage is the inverse of MP.

^4^For example, in all 2-loci Y-STR haplotype combinations (total 16 × 15 ÷ 2 = 120), the combination DYS458 and DYS385 ('5, 15' in the second row) had the lowest MP.

Clearly, Y-STR loci are not as good as autosomal STRs for single-source profile comparisons, and as the current battery of autosomal STRs is sufficient for large database searches, there would be no need to include a set of Y-STRs. However, Y-STRs are very good for kinship analysis and for power of exclusion in familial searching and missing-persons identification. At this time Y-STR loci are not included in reference profiles (other than for missing persons) in the CODIS database, thus a familial search candidate list requires substantial work by the laboratory to eliminate a number of candidates. Currently, the DNA of familial search candidates is retrieved and typed for Y-STRs, and samples with non-matching Y-STR profiles are excluded. Substantial labor is required, and turnaround times can be slow. Faster turnaround times for investigative leads could be achieved if the new core loci included several Y-STRs instead of adding more autosomal loci. Indeed, only a small number of Y-STR loci are needed (probably only around 6).

Y-STR haplotypes can also be useful in interpretations of mixtures, especially when a single male DNA is mixed with female DNA. Ge *et al. *[27] estimated the power of exclusion of 16 Y-STR haplotypes with a relatively small database size, and found that 95% of 2-person mixtures had 10 or fewer candidate haplotypes in the database. Further studies need to be carried out with fewer Y-STRs (around 6) in a larger Y-STR database to estimate the power of exclusion and number of possible contributors with using solely Y-STRs. The Y-STRs could then be combined with autosomal STRs for further evaluation. Consideration should include the effect of maintaining the current autosomal STR systems (that are in extant commercial kit formats) and of combining them with five or six informative Y-STRs. Increasing the number of investigative leads should be a primary motivation of the core loci selection.

### Combining autosomal Y-chromosome short tandem repeats

As described in the two sections above, both autosomal STRs and Y-STRs have their places in forensic applications. Combining both autosomal STRs and Y-STRs may best meet the needs of forensic applications for single-source and kinship searches in large databases. Thus, we evaluated the performance of a combination of autosomal STRs and Y-STRs when the total number of core loci is limited because of the quality and quantity of forensic DNA. The loci in section B were not included because of their limitations in independence, MP, and/or mutation rates.

**Figure 3:**
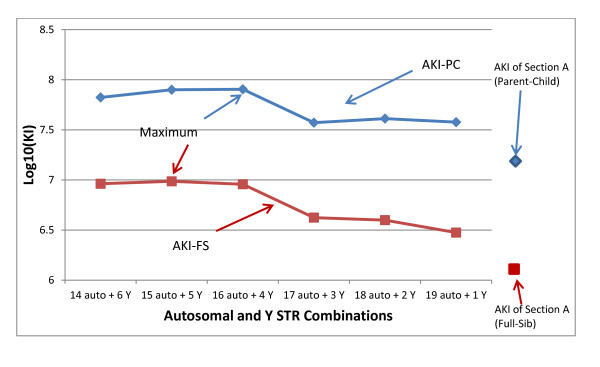
**The log_10 _of the average kinship index (AKI) distributions of full-sibling and parent/child relationships**. Log_10_(AKI) distributions of full-sibling and parent/child relationships with the most informative autosomal and Y-chromosome STRs (Y-STRs) in Tables 1 and 4. The horizontal axis labels are '14 auto- + 6 Y-STRs' to '18 auto- + 2 Y-STRs' which are the combinations of a specified number of the most informative autosomal STRs in section A (except for the D12S391 locus, because this locus is linked with the VWA locus) and a specified number of the most informative Y-STRs. The term '19 auto- + 1 Y-STRs' refers to all 19 autosomal STR loci in section A and the most informative Y-STR (DYS385). Independence between D12S391 and VWA was assumed in calculation of AKI values of '19 auto- + 1 Y-STRs'. In all calculations, the D5S818 and CSF1PO loci were assumed to be independent (although current data do not support the assumption). The true AKI of '19 auto- + 1 Y-STRs' should be slightly lower.

We calculated the AKI of parent/child and full-sibling relationships for combining various numbers of autosomal STRs and Y-STRs out of 20 total loci (Figure 3). The AKI values of 19 autosomal STRs from section A and the single most informative Y-STR (DYS385) were lower than other combinations with more Y-STRs (and would be even lower if the DYS391 locus was included instead of the DYS385 locus). For illustrative purposes, independence was assumed for all autosomal loci and for the Y-STR haplotypes with the autosomal loci. The true AKI values of 19 autosomal- + 1 Y-STR loci should be slightly lower, because both the D12S391 and VWA loci were included, and for estimation purposes were assumed independent although it is known that they are not. Assuming independence for the other syntenic pair (D5S818 and CSF1PO) may also change the AKI slightly. The maximum AKI values were found for the combination of 16 autosomal + 4 Y-STRs for parent/child, and of 15 autosomal- + 5 Y-STRs for full-sibling relationships. The AKIs of 14 autosomal- + 6 Y-STRs were comparable with the maximum values, but the combinations with 1, 2 or 3 Y-STR loci had apparently lower AKIs. The AKIs of section A loci were 1.47 × 10^7 ^and 1.16 × 10^6 ^for parent/child and full-sibling, respectively, which are apparently lower than those of 16 autosomal- + 4 Y-STRs, 15 autosomal- + 5 Y-STRs, and 14 autosomal- + 6 Y-STRs. Although the curves may vary with the population, the Caucasian population data example shows that the combinations of 16 autosomal- + 4 Y-STRs, 15 autosomal- + 5 Y-STRs, and 14 autosomal- + 6 Y-STRs may be good options for kinship analysis (and probably mixtures, although these simulations were not carried out in the present study). An analysis can be performed to include recombination fractions, but the general trends of the AKI distributions will not change.

For single-source profile comparisons (Table 6), the combination of 16 auto- + 4 Y-STRs has an MP of 1.53 × 10^-21^. The current commercial kits with the five most informative Y-STRs also can yield an MP of at least 7.74 ×10^-20^. The MP values of these tested autosomal STR and Y-STR combinations are sufficiently low to minimize adventitious hits in large database searches. If the CODIS and, for example, European databases choose the same 5 most informative Y-STRs, plus the current shared 7 autosomal loci, the MP of shared loci between the USA and Europe can reach 5.37 × 10^-12^. Kinship analysis may even be practical in international data exchange with these extra shared 5 Y-STRs. (Note: we are not recommending that Europe should adopt several Y-STRs for compatibility; Europe already has selected a core set and has added some mini-STRs for more successful typing of degraded samples. We simply provide the data to indicate that there are additional considerations for selecting core loci.) Further studies are needed to estimate the false inclusion and exclusion rates for identifying most common relationships, and their applicability in interpreting mixture profiles. Once the data are obtained, more informed decisions can be made for selecting core loci.

**Table 6:** Match probabilities (MPs) of short tandem repeat (STR) loci combinations.

STR combinations	MP
14 auto + 6 Y	1.53 × 10^-21^
15 auto + 5 Y	2.42 × 10^-22^
16 auto + 4 Y	4.48 × 10^-23^
17 auto + 3 Y	9.64 × 10^-23^
18 auto + 2 Y	4.83 × 10^-24^
19 auto + 1 Y^1^	3.38 × 10^-25^
Section A	9.20 × 10^-25^

Identifiler + 5 Y	7.74 × 10^-20^
PowerPlex16 + 5 Y	3.34 × 10^-20^
NGM + 5 Y	2.21 × 10^-21^

7 shared auto + 5 Y^2^	5.37 × 10^-12^

11 shared auto + 5 Y^3^	6.82 × 10^-16^

6 shared auto + 5 Y^4^	6.71 × 10^-11^

^1^The Y- chromosome STR in this row is the DYS385 locus, not the DYS391 locus in section A.

^2^Between 13 CODIS core loci and European loci.

^3^Between USA and China.

^4^Between China and Europe.

## Discussion

The purpose of creating criminal DNA databases is to generate investigative leads. With the growth of databases and expansion of applications, adding more STR loci into databases has been proposed or discussed in the USA [2], Europe [4-6], and China [24]. Additional and alternative loci are being proffered. We promote the review of the current state of the art, and welcome recommendations for the future potential of the art. We have provided some example analyses for illustrative purposes for decision- and policy-makers and stakeholders to consider, beyond those considered by Hares [2]. Such decisions have an important influence on developing investigative leads and could cost millions of dollars. Thus, judicious decisions with community input should be sought. The current battery of loci performs well for some applications, but is not sufficient for others. However, increasing the number of autosomal STR loci may not be the only or the best solution. For overall applications, a small set of Y-STRs with the current STR batteries may be more practical, especially if analyses for kinship (including familial searching), and possibly for mixtures, are to be part of the process. Indeed, a combination of autosomal and Y-STRs will perform well for single-source searches. The analyses described here should be expanded with larger simulations and include other relevant populations to generate data for more informed decision-making.

We strongly urge that the selection process consider casework applications as the primary driving force in the selection of core loci. The quantity and quality of DNA derived from casework evidence will always be a limiting factor. For instance, if the current loci are being reconsidered, the performance of large amplicon loci should be evaluated, especially in light of expanded analyses on forensic evidence, such as 'touch DNA'. For example, the FGA locus may provide a high discrimination power, but its performance in challenged samples may be poor compared with some less informative but smaller-sized amplicon loci. Partly the performance is due to amplicon size limitations, and partly to the wide spread of the FGA alleles. Data on success rates for the various loci (obviously in kit format) should be collected for forensic-evidence analyses.

The potential increase in resource strain on laboratories must also be weighed against the gain in power. Given the direction of casework towards typing more challenging samples (such as low-quantity and/or degraded samples), those STR loci that can be converted to mini-STRs might be considered the most desirable and thus it might be better to consider rejecting loci that cannot be converted to mini-STRs. Additionally, the FBI Working Group may have been too narrow in its STR performance review. For example, we have already pointed out that the PentaD locus, relegated to section B, is more informative than several of the STR loci in section A. However, the largest allele in the PentaD allelic ladder is a 17. Thus, it is entirely feasible that the PentaD locus (and the PentaE locus) could be converted to a mini-STR locus.

Indeed, a multiplex kit has reportedly been developed with the amplicon size of the Penta loci reduced [28]. Perhaps the selection criteria should take into account the potential size of amplicons and avoid being constrained by current kit designs. CODIS could possibly drive the development of mini-STR configuration kits. In addition, developing very large multiplex kits may be possible for reference samples, but may be less easily met for casework demands. Sensitivity of detection is paramount for casework kits. Thus, the requirement for more loci may translate into two kits, putting greater demand on the casework laboratories and possibly still not increasing the number of typed loci if the DNA evidence is compromised. If more loci are to be added, it may be better to add more Y-STR loci instead of only autosomal loci, as the Y-STR loci (in concert with the core loci) can support both direct and indirect comparisons effectively. Using the criterion of casework performance, the conclusions for loci to include and exclude in a core set may change from those proffered by Hares [2].

Low-level population substructure is another criterion for a good forensic locus. Population substructure is usually measured by Fst (i.e., inbreeding coefficient). High Fst can reduce the information content of the locus. The National Research Council (NRC) Report II [29] recommended a conservative Fst value of 0.01 for major populations. As they have multiple alleles per locus and are highly polymorphic, the most commonly used autosomal STR loci are expected to have a low average Fst. Although the effect is small if a couple of higher Fst loci are added to a core set, it would be desirable to have population data from major populations to test for substructure effects before selecting loci. Similarly, it would be desirable to generate mutation-rate data before selecting loci. Population studies will be difficult to achieve in the current forensic arena because sufficient population data will not be generated by forensic laboratories unless the loci are part of a core set or in commercial kits. Funding could be provided to support CODIS endeavors to ensure a robust and long-lasting system is developed.

## Conclusion

Assessing the CODIS loci is a laudable endeavor that needs to be carried out. We did not undertake all the studies necessary to evaluate the current loci and the needs that these proposed loci should meet. However, based on the discussion and simplified studies given here (generated for illustrative purposes), there are several points to consider.

The use of mitochondrial DNA was not considered in these studies because most of the profiles in CODIS are from men, and different methods or technology would be required for mtDNA typing. However, the database (and other databases worldwide) continues to grow, and proportionally more women (and maternal associations) may populate the database in the future. Therefore, future discussion should consider the value of some mtDNA markers for CODIS applications. Other markers that might be discussed and evaluated for long-term benefit include single-nucleotide polymorphisms (to include indels) and X-STRs. Next-generation sequencing technologies may make it possible to type autosomal STRs, Y-STRs, mtDNA and single-nucleotide polymorphisms in one analysis, and technical capability projections might be considered. Additionally, we did not address the effect of the selection criteria under moderate-stringency search parameters, or whether markers should be in the public domain. To better serve the lofty goals of improving single-source profile comparisons, mixture comparisons, kinship analyses such as missing-persons identification and familial searching, and international data sharing, more comprehensive studies are required to provide sufficient information to the decision-makers and stakeholders about constructing a new set of core loci for CODIS. Finally, the need to improve typing capabilities for casework analyses, and especially challenged forensic samples, must be the primary criterion for selecting core loci for CODIS. The most polymorphic loci will tend to be better for mixture deconvolution, but will tend to have higher mutation rates. These loci also will have the greatest spread of alleles, and thus be more subject to degradation. Therefore, a balance may need to be sought between information content and allele spread. We contend that most currently used STR loci that can be converted to small-sized amplicons will perform better overall for challenged casework and still be useful for mixture deconvolution (even if they are not the most polymorphic of loci) and for kinship analyses (because they will tend to have lower mutation rates).

## List of abbreviations

CODIS: Combined DNA Index System; FS: full sibling; AKI: average kinship index; EMP: expected match probability; KI: kinship index; MP: match probability; NDIS: National DNA Index System; PC: parent/child; STR: short tandem repeat.

## Competing interests

The authors declare that they have no competing interests.

## Authors' contributions

JG carried out the analysis design, data analyses, and data interpretation, and took the lead in writing the manuscript. BB oversaw the study, and was involved in analysis design, data interpretation, and writing the manuscript. AE was involved in discussions of certain issues, and contributed to manuscript drafting. All authors read and approved the final manuscript.
